# Supplementary material for: Fluxing of mantle carbon as a physical agent for metallogenic fertilization of the crust
Source: Nat Commun. 2020 Aug 28;11:4342. doi: 10.1038/s41467-020-18157-6 (PMC7455710; doi:10.1038/s41467-020-18157-6)
Supplement: Supplementary file 2 — Description of Additional Supplementary Files [file 41467_2020_18157_MOESM2_ESM.pdf]

Description of Additional Supplementary Files

File Name: Supplementary Data 1
Description: Carbon and oxygen isotope data for carbonate minerals from the Valmaggia and Sron Garbh intrusions, and country rocks in the Ivrea Zone.
